# Prevalence and distribution of regional scar in dysfunctional myocardial segments in Duchenne muscular dystrophy

**DOI:** 10.1186/1532-429X-13-20

**Published:** 2011-03-11

**Authors:** Kenneth C Bilchick, Michael Salerno, David Plitt, Yoav Dori, Thomas O Crawford, Daniel Drachman, W Reid Thompson

**Affiliations:** 1 Department of Medicine, University of Virginia Health System, Charlottesville, Virginia, USA; 2 Department of Pediatrics, Johns Hopkins Medical Institutions, Baltimore, Maryland, USA; 3 Department of Neurology, Johns Hopkins Medical Institutions, Baltimore, Maryland, USA

## Abstract

**Background:**

The *segmental *relationship between cardiovascular magnetic resonance (CMR) peak circumferential strain (Ecc) and myocardial scar has not been well characterized in Duchenne muscular dystrophy (DMD), and it is unknown whether echocardiography accurately measures Ecc in DMD. We assessed segmental Ecc and scar using CMR with myocardial tissue tagging and late gadolinium enhancement (LGE) in patients with DMD, then compared CMR with echocardiographic velocity vector imaging (VVI) for regional Ecc based on independent observer assessments.

**Results:**

Participants enrolled (n = 16; age 8-23) had median left ventricular (LV) ejection fraction of 0.52 (range 0.28-0.69), and 156 basal and mid-cavity myocardial segments from the 13 patients completing the LGE protocol were analyzed for strain and scar. Segmental CMR Ecc in the most negative quartile (quartile 4) ruled out scar in that segment, but scar was present in 46% of segments in the least negative (most dysfunctional) Ecc quartile 1, 33% of Ecc quartile 2 segments, and 15% of Ecc quartile 3 segments. Overall scar prevalence in inferior, inferolateral, and anterolateral segments was eight times higher than in inferoseptal, anteroseptal, and anterior segments (p < 0.001). This increased proportion of scar in lateral versus septal segments was consistent across CMR Ecc quartiles (quartile 1: 76% versus 11%, p = 0.001; quartile 2: 65% versus 9%, p < 0.001; quartile 3: 38% versus 0%, p < 0.001). Echocardiographic analysis could be performed in 12 of 14 patients with CMR exams and had to be limited to mid-cavity slices. Echo segmental Ecc in the most negative quartile made scar by CMR in that segment highly unlikely, but the correlation in segmental Ecc between CMR and echo was limited (r = 0.27; p = 0.02).

**Conclusions:**

The relationship between scar and Ecc in DMD is complex. Among myocardial segments with depressed Ecc, scar prevalence was much higher in inferior, inferolateral, and anterolateral segments, indicating a regionally dependent association between abnormal Ecc and scar, with free wall segments commonly developing dysfunction with scar and septal segments developing dysfunction without scar. Although normal echocardiographic Ecc predicted absence of scar, regional echocardiographic Ecc by VVI has only a limited association with CMR Ecc in DMD.

## Background

Duchenne muscular dystrophy (DMD), the most common of the muscular dystrophies, with an incidence of 1 in 3,500 males, is an X-linked recessive disorder resulting from a disabling mutation of the gene encoding dystrophin, a sarcolemmal protein found in skeletal and cardiac muscles [1,2]. There is progressive skeletal muscle weakness with loss of ambulatory ability in the teenage years. Death is usually due to cardiac or respiratory failure [3,4], and distinctive pathologic findings have been noted [5,6]. With improvements in overall management and respiratory treatment [7], there has been increasing interest in the prevention and treatment of cardiac disease in DMD [8].

Cardiovascular magnetic resonance (CMR) has recently been investigated as a means to characterize the cardiac abnormalities of DMD [9-12]. In addition to measuring cardiac function accurately, CMR with myocardial tissue tagging (MTT) [13,14] and late gadolinium enhancement (LGE) [15] accurately assesses circumferential strain (Ecc) and scar, the latter being associated with increased arrhythmia risk and poor cardiac outcomes [16-18] in heart failure.

The prevalence and distribution of regional myocardial scar relative to regional Ecc in DMD is not well understood and has important implications for our understanding of myocardial dysfunction in DMD. Ecc is a sensitive indicator of occult myocardial dysfunction in DMD [9], and average mid-cavity Ecc has been shown to deteriorate with age in DMD [11,12]. In one study of DMD and Becker muscular dystrophy patients, regional myocardial scar was compared with qualitative regional function as assessed by visual inspection [10], but not with regional Ecc. In the present series, we provide a detailed segment-by-segment comparison of Ecc and scar in DMD patients with a broad range of age and global left ventricular (LV) function to address the hypothesis that the strength of the association between depressed segmental Ecc and scar in DMD depends on the myocardial region being evaluated.

The study also sought to assess whether velocity vector imaging (VVI) assessment of Ecc would have some utility in DMD. VVI echocardiography offers far more advanced technology than that used in the original echocardiographic studies in DMD patients [19]. VVI assesses Ecc from echocardiographic short-axis images and has been studied in both adults and children referred for cardiac resynchronization therapy [20,21], as well as patients after myocardial infarction [22]. Although CMR is considered the gold standard for assessment of myocardial scar and regional Ecc, a complete CMR examination with LGE and regional strain assessment may be difficult for some patients with DMD due to reasons such as respiratory insufficiency, severe contractures, intolerance of recumbent immobility for the time necessary, difficulties with intravenous access, and limited capacity for cooperation if there is impaired cognition. Traditional echocardiographic evaluation in DMD is known to be limited by suboptimal imaging windows, but whether VVI provides useful information in selected patients with DMD has not been determined.

## Methods

### Enrolment of participants

The protocol was approved by the Johns Hopkins Institutional Review Board and carried out in the General Clinical Research Center of the Johns Hopkins Hospital.

### CMR protocol

All patients underwent CMR studies using a 1.5-T clinical scanner (Magnetom Avanto, Siemens, Malvern, PA) with a phase array receiver coil on the chest. Cine images were acquired using a steady-state free precession pulse sequence with the following parameters: temporal resolution: 40 ms, echo time: 1.27 ms, flip angle: α = 50°, field of view: 36 cm, slice thickness: 8 mm, matrix size: 256 × 161, and bandwidth: 930 Hz/pixel.

Myocardial tagging was performed using an electrocardiogram-triggered spoiled gradient echo pulse sequence with spatial modulation of magnetization grid-tagging. Short-axis slices were acquired at the left ventricular base, mid-cavity, and apex with the following parameters: repetition time: 3.5-7.2 ms, echo time: 2.0-4.2 ms, flip angle: α = 12°, field of view: 40 cm, slice thickness: 8 mm, matrix size: 256 × 140, bandwidth: 275 Hz/pixel, and tag spacing: 7 mm.

LGE images in locations identical to the cine images were acquired 10-15 minutes after a bolus of 0.2 mmol/kg gadodiamide (Omniscan, GE Healthcare, Buckinghamshire, United Kingdom) or gadopentetate dimeglumine (Magnevist, Bayer Healthcare Pharmaceuticals, Tarrytown, New York) in all patients. An inversion recovery fast gradient-echo pulse sequence was used for the acquisition with the following parameters: repetition time: 5.4 ms, echo time: 1.3 ms, flip angle: α = 20°, field of view: 36-40 cm, slice thickness: 8 mm, matrix size: 256 × 192, and inversion recovery time: 250 ms (adjusted to null the signal of normal myocardium).

### Strain and volumetric analysis

Short axis tagged slices were analyzed in blinded fashion by the harmonic phase (HARP) method to assess strain (Diagnosoft, Cary, NC). Segmental peak systolic strains were determined in 24 segments for basal and mid left ventricular slices. These were then grouped and averaged to provide average peak strain values for each of 6 segments in basal and mid-cavity slices (anteroseptal, anterior, anterolateral, inferolateral, inferior, inferoseptal). Thus, for each patient, we report the peak circumferential systolic strain in six myocardial regions at both basal and mid-cavity myocardium. Left ventricular end-systolic volume (LVESV) and left ventricular end-diastolic volume (LVEDV) were measured, and left ventricular ejection fraction (LVEF) calculated for each study patient. The strain analysis was performed by two experienced investigators (KCB, DP), and interobserver variability for segmental strain was determined for 60 segments (30 basal and 30 mid-cavity segments) acquired from 5 study participants (12 segments/participant).

### Scar analysis

LGE images were analyzed, and abnormally enhanced myocardium was determined based on prior methods [17,23] at six circumferential regions in both basal and mid-cavity slices, corresponding to the segments that had been assessed for strain. The transmural extent of scar was determined based on established methodology [16] using the percent of total myocardial thickness at each segment (no scar, 1-25%, 26-50%, >50%). Left ventricular scar volume was determined as a percentage of left ventricular volume, as previously described [24]. Segmental scar was evaluated by two independent observers (KCB, MS).

### Echocardiographic analysis

2-dimensional echocardiographic images were acquired using either GE Vivid 7 or Siemens Acuson machines and stored digitally at 30 frames per second (Syngo, Siemens). On a separate workstation (Syngo Workplace, Siemens), VVI (Velocity Vector Imaging, Siemens) was used to determine peak Ecc. The endocardial border was manually traced at end-systole in the parasternal short axis view at the level of the papillary muscles, corresponding to the mid-cavity left ventricle, by a single researcher (WRT) without knowledge of the CMR data. A single cardiac cycle was selected for analysis. The software generated a vector for each frame in the cardiac cycle at 48 points along the endocardial border and calculated Lagrangian strain at each location. MATLAB (The MathWorks, Inc.) was used to find the peak (most negative) strain value for each point, and the average peak values from 8 consecutive points in each of the six segments in the mid-cavity left ventricle were calculated.

### Neuromuscular evaluation

All but one subject had quantitative hand-held myometry evaluation of bilateral elbow and knee flexion and extension strength within two months of the time of cardiac evaluation. Strength was determined in units of pounds as the average of these myometry measurements for muscle groups associated with the elbow and knee. An overall composite strength score of 0 indicates that patients were not capable of any movement in these muscle groups, although they may have some strength in the distal extremities.

### Statistical methods

Continuous and categorical variables were reported as the median and interquartile range (IQR). Chi square tests and Fisher exacts tests were used to compare proportions among groups. We used t-tests for comparisons between continuous strain variables. Linear regression was used to determine associations between selected continuous variables, with the Pearson correlation coefficient (r) and p-values reported. The intraclass correlation coefficient (ICC) and associated 95% confidence interval were determined for the analysis of interobserver variability of segmental strain (SAS 9.2, SAS Institute Inc.), as previously reported [25].

## Results

### Overall characteristics of study population

16 DMD subjects were enrolled (age range 8-23), with individual clinical characteristics shown in Table 1. The table shows that patients over a broad age range were included and had varying degrees of neuromuscular impairment. As expected, myometry-based strength measurements tended to worsen with age (r = -0.68; p < 0.01), with very significant limitations observed for some patients. LVEF also varied significantly in these patients from 0.28 to 0.69.

**Table 1:** Baseline characteristics

Age	Years in Chair	Years on Steroids	Myometry Strength (pounds)*	ECG R/S Ratio V1
8	0	6	.	0.6

9	0	8	14.3	3.0

10	1	3	10.3	1.6

11	0.5	6	6.3	1.0

12	3	0	0	0.6

12	0	6	14.8	0.7

13	2	5	12.8	1.6

13	0	2	2.0	0.8

15	8	0	0	0.5

15	6	8	1.0	0.9

16	5	7	3.0	1.0

17	8	15	0.3	2.0

20	12	0	0	2.4

20	3	0	0	0.5

22	12	1	0	0.8

23	8	18	0.5	0.8

* Indicates average myometry readings for muscle groups associated with the knee and elbow

Although echo studies were performed in all patients, two patients (ages 12 and 13) could not tolerate the CMR due to limited cooperation in one and contractures that prevented positioning in the MRI chamber in the second, and LGE could not be performed in another patient due to difficulty with venous access. Thus a total of 14 subjects had CMR, and 13 had LGE studies. Scar was rare in apical segments, so the analysis was performed in the basal and mid-cavity segments for Ecc and scar, based on a 12-segment model (equivalent to the basal and mid-cavity segments of a standard 17-segment model). Comparative analyses of scar and strain were performed both at the level of individual segments (n = 156) and on the basis of individual patients.

### CMR analysis of regional strain and scar

**Figure 1:**
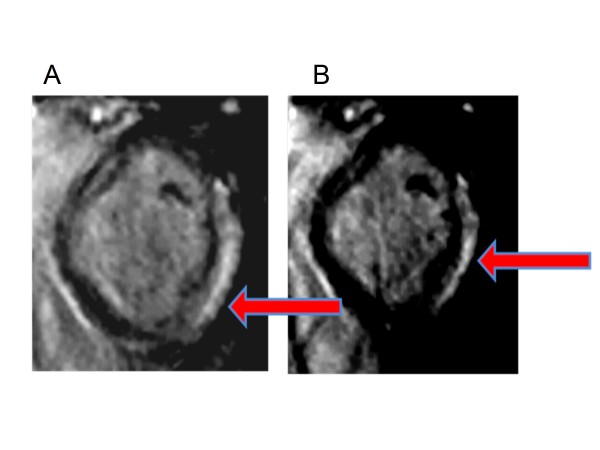
**DMD late gadolinium enhancement**. Basal (panel A) and mid-cavity (panel B) slices of subepicardial and midmyocardial scar involving inferolateral and anterolateral segments in a patient with DMD. The white (hyperenhanced) region (arrow) is scar, while the black represents normal myocardium.

**Figure 2:**
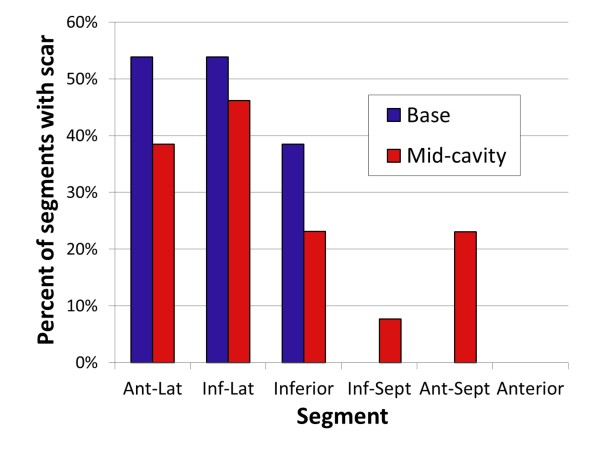
**Distribution of myocardial scar in basal and mid-cavity segments**. The bar graph shows inferolateral, inferior, and anterolateral predominance of scar, as well as the distribution of scar between basal and mid-cavity segments. Ant-Lat = anterolateral; Inf-Lat = inferolateral; Inf-Sept = inferoseptal; Ant-Sept = anteroseptal.

For the segmental analysis, 156 segments were analyzed for scar and Ecc by CMR. Aggregate results for scar and average Ecc by patient are shown in Table 2. There was very good interobserver agreement for Ecc, with an intraclass correlation coefficient of 0.94 [0.90-0.96], consistent with the previously high reported reliability of Ecc measurements derived from MTT [25]. In addition, there was also a 95% agreement between independent observers with respect to whether segments did or did not have scar. Scar typically involved at least the subepicardium with a predominant distribution in inferior, inferolateral, and anterolateral segments, as shown in the example in Figure 1. 24% of segments had scar present by LGE with about a third each having 1-25% transmurality, 26-50% transmurality, and greater than 50% transmurality, respectively. Scar was present in both basal and mid-cavity segments, as shown in Figure 2. 89% of segments with any detectable scar were either inferior, inferolateral, or anterolateral segments (group 1), and the remaining 11% of segments with scar were either inferoseptal, anteroseptal, or anterior segments (group 2); therefore, scar was eight times more common in group 1 (lateral) segments compared with group 2 (septal) segments (p < 0.001). Of note, patients with scar in septal segments also had significant scar involving inferolateral segments.

**Table 2:** Overall Summary of CMR findings

	Median	IQR
LVEF	0.52	(0.45, 0.57)
LVEDV (cc)	68	(64,97)
CMR Base Ecc (%)	-13.3	(-10.0,-16.4)
CMR Mid-cavity Ecc (%)	-13.7	(-11.0,-16.9)
Echo Mid-cavity Ecc (%)	-15.0	(-9.9,-19.8)
Percent Scar Volume (%)	6	(0,10)

IQR = Interquartile Range

**Figure 3:**
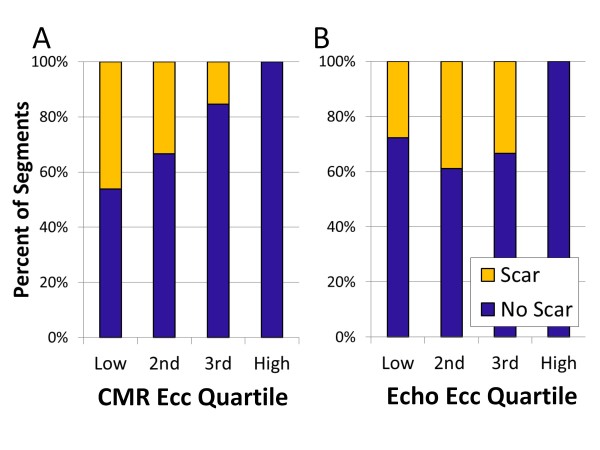
**Segmental scar and circumferential strain by CMR versus VVI echocardiography**. The distribution of myocardial fibrosis by quartile of Ecc as assessed with (A) CMR (all segments) or (B) VVI echocardiography (mid-cavity segments only) is shown. A) No segments with Ecc by CMR in the fourth (most negative) quartile had myocardial scar. Myocardial scar was most common in the two lowest (most dysfunctional) Ecc quartiles. B) Scar was also unlikely in segments falling into the fourth quartile of Ecc as assessed by echo VVI and was distributed in the lower three echo Ecc quartiles. 1^st ^quartile = least negative Ecc (most dysfunctional); 4^th ^quartile = most negative Ecc.

**Figure 4:**
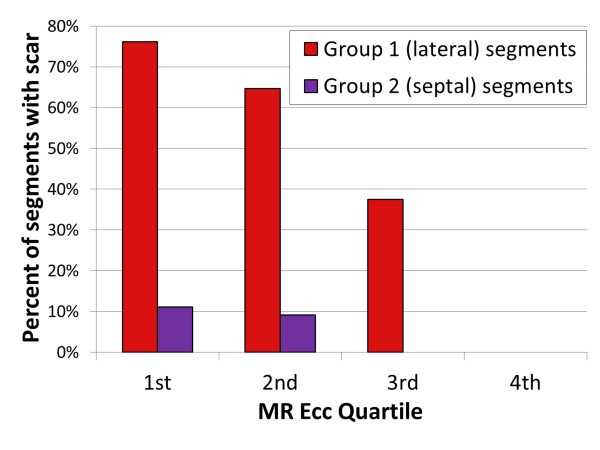
**Increased proportion of scar in lateral versus septal segments across CMR circumferential strain quartiles**. The figure shows an increased proportion of scar in group 1 (lateral) segments versus group 2 (septal) segments based on the analysis of all 156 myocardial segments. This effect was consistent across all lower three CMR quartiles of Ecc, indicating that there is increased prevalence of segmental scar at any level of segmental Ecc in group 1 (lateral) segments versus group 2 (septal) segments. In other words, the association between depressed Ecc and scar has a significant regional dependence, with dysfunctional lateral segments much more likely to have scar than dysfunctional septal segments. 1^st ^quartile = least negative Ecc (most dysfunctional); 4^th ^quartile = most negative Ecc.

The mean Ecc for segments in groups 1 and 2 were similar (p = NS). However, the relationship between segmental Ecc and scar was complex. As shown in Figure 3A, CMR Ecc in the highest quartile (quartile 4, most negative) for all segments excluded scar. Segments in Ecc quartile 1 (least negative, most abnormal strain) had the highest proportion (46%) with evidence of LGE, while 33% of Ecc quartile 2 segments and 15% of quartile 1 segments had evidence of scar by LGE (p < 0.001 for differences in scar prevalence by quartile). Figure 4 shows an increased proportion of scar in group 1 (lateral) segments versus group 2 (septal) segments that was consistent across CMR Ecc quartiles (quartile 1: 76% versus 11%, p = 0.001; quartile 2: 65% versus 9%, p < 0.001; quartile 3: 38% versus 0%, p < 0.001). These findings are consistent with a regionally dependent strength of association between depressed segmental Ecc and scar.

**Figure 5:**
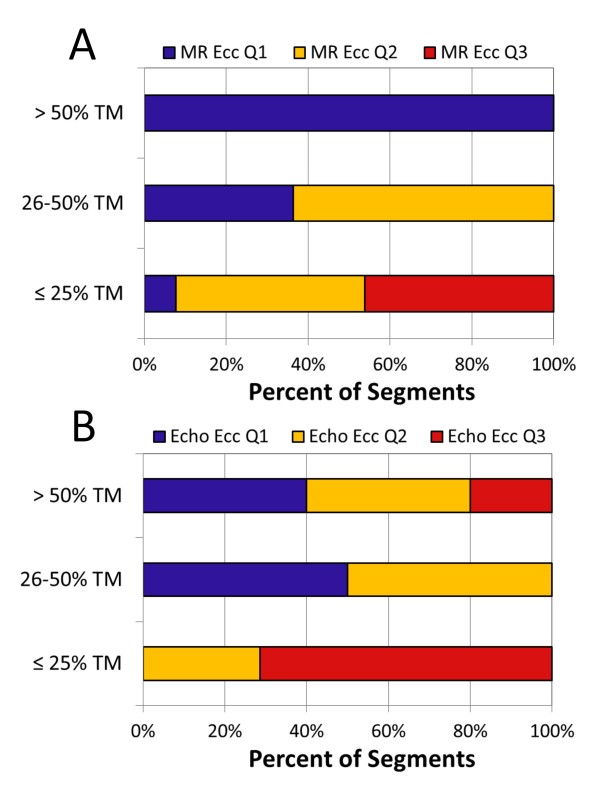
**Relationship between segmental transmurality of scar and regional Ecc**. The relationship between the segmental transmurality of scar (characterized as 0-25% transmural, 26-50% transmural, or >50% transmural) and regional Ecc is shown, with regional Ecc characterized by (A) CMR (all segments), and (B) VVI echocardiography (mid-cavity segments only). Note that all segments with scar of greater than 50% transmurality were in the most dysfunctional CMR Ecc quartile (Q1), and segments with intermediate scar transmurality were distributed among the lower two Ecc quartiles (Q1 and Q2).

The relationship between the transmurality of scar and circumferential strain by CMR and echocardiography is shown in Figure 5. Panel A describes the relationship between scar transmurality and CMR-based Ecc. Segments with the most transmural scar were in the lowest quartile of Ecc (quartile 1) by CMR, while segments with intermediate scar transmurality were distributed between Ecc quartiles 1 and 2. With respect to echocardiography, segments with more transmural scar tended to be in the lowest two echo Ecc quartiles (quartiles 1 and 2), while segments with the least transmural scar corresponded mostly to echo Ecc quartile 3.

### Echocardiographic analysis relative to CMR findings

VVI analysis for regional Ecc by echo could be performed in only 12 of the 14 patients who had CMR and only in mid-cavity rather than basal segments. As shown in Table 2, the medians for echo and CMR mid-cavity Ecc were similar; however, the correlation between echo and CMR assessments of segmental Ecc was limited (r = 0.27; p = 0.02). As shown in Figure 3B, segments with echo Ecc in the highest quartile predicted absence of scar, whereas segments with scar were distributed in the lower three (more dysfunctional) quartiles of echo Ecc.

### Overall measures of function relative to scar burden

54% of patients had evidence of late gadolinium enhancement in at least one segment, and in all patients with scar the abnormality involved at least the lateral segments. Those with scar had a mean percent scar volume of 13% (range 6-29%). There was an inverse correlation between LVEF by CMR and the percent scar volume (r = -0.68, p = 0.01). Although scar was common in patients with moderately and severely reduced LVEF, some patients with only mildly reduced LVEF also were noted to have scar. Percent scar volume also correlated with average peak CMR strain in basal (r = 0.75, p < 0.01) and mid-cavity (r = 0.74, p < 0.01) segments.

### Electrocardiographic findings

Analysis of the 12-lead ECG was performed for all patients. All patients had an R wave in lead V1 with amplitude ranging from 4-19 mV. Small q waves were present in lead I and aVL in most patients, but only one patient had prominent Q waves in leads I and aVL. Several ECG parameters were examined for prediction of inferior/lateral scar, including the R wave amplitude in V1, the R/S ratio in lead V1, and Q wave presence in leads I and aVL, but none demonstrated significant predictive value for scar. R/S ratios in V1 for individual patients are shown in Table 1.

## Discussion

The important findings of this study include the following: 1) segmental basal or mid-cavity Ecc by CMR in the highest quartile makes regional myocardial scar highly unlikely; 2) segmental scar is much more common in inferolateral, anterolateral, or lateral segments with depressed Ecc than in inferoseptal, anteroseptal, and anterior segments with depressed Ecc, consistent with a regionally dependent strength of association between depressed segmental Ecc and scar; 3) echocardiographic segmental mid-cavity Ecc based on velocity vector imaging in the highest quartile also makes scar (by CMR) in the corresponding segment unlikely, but there was only a limited correlation for mid-cavity segmental Ecc assessed using the two modalities.

The importance of assessing and understanding myocardial scar and function in DMD has taken on increased clinical relevance with recent evidence that medical therapy with antifibrotic agents such as angiotensin converting enzyme inhibitors [8], angiotensin receptor blockers [26], and membrane sealants [27] may help prevent or slow the progression of cardiac disease in DMD. Our findings regarding scar are consistent with findings from earlier autopsy studies in DMD showing that myocardial fibrosis initially involves the "posterobasal" epicardium, progresses to involve the epicardial half of the LV free wall, then occasionally involves septal segments as the fibrosis becomes more severe and more transmural [5].

Although autopsy studies and CMR both provide excellent assessments of myocardial scar, CMR has the advantage of also providing quantitative regional assessment of myocardial function with Ecc. Of note, circumferential strain has been better studied in DMD than radial or longitudinal strain [9,10]. Ecc is also particularly well suited for evaluation of affected LV segments in DMD because left ventricular myofiber orientation is predominantly circumferential [28], and its use has been established for mechanical function in heart failure [29,30].

As in prior autopsy studies, we found that scar predominantly involved LV free wall segments and that septal segments were involved less frequently and only in patients with significant LV free wall involvement. In addition, the present study shows that there were similar numbers of LV septal and free wall segments with depressed Ecc; however, the septal segments with abnormal Ecc values were much less likely to have myocardial scar than free wall segments with similarly abnormal Ecc values. Although the pathophysiologic mechanism behind this curious tendency for the septal segments to become dysfunctional without scar and LV free wall segments to become dysfunctional with scar is unknown, TGF-β has emerged as the major likely effector of fibrosis in DMD. TGF-β inhibits terminal differentiation of myoblasts and is associated with the development of peripheral muscle fibrosis in DMD [31]. Losartan (an angiotensin II receptor blocker) has been shown to counteract the effects of TGF-β, decrease muscle fibrosis, and improve muscle regeneration in *mdx *mice [32]. In addition, perindopril (an angiotensin converting enzyme inhibitor) has been shown to decrease the risk of developing LV systolic dysfunction in patients with DMD when started at a young age [8]. Although it is unknown if and how regional differences in these molecular pathways result in differential regional LV wall fibrosis in DMD, it has been shown that regional differences in LV wall mechanics can result in differential expression of genes related to matrix remodeling and hypertrophy in other cardiac disease states [33].

Our findings regarding Ecc and scar complement those reported by Silva et al in a series of 10 DMD or Becker muscular dystrophy patients with regional dysfunction assessed by visual inspection rather than Ecc [10]. In the series of Silva et al, a small number of segments with apparently normal function had positive LGE, indicating that the prevalence of scar in segments with normal segmental function depends on whether visual inspection or Ecc is used to define normal function.

Of note, we analyzed Ecc in this study using the well-validated HARP method [13] and demonstrated good interobserver agreement for Ecc, consistent with the demonstrated reliability of this method for Ecc analysis [25]. Furthermore, our average and range for Ecc were consistent with what has been previously reported. For example, the median mid-cavity peak Ecc was within 1% of the mean Ecc reported in a recent series [34]. In addition, our range for mid-cavity Ecc was consistent with the range for mid-cavity Ecc reported in a series of young DMD patients [9].

With respect to VVI echocardiography, adequate echocardiographic strain data could not be obtained in all DMD patients due to limited echocardiographic windows, and the analysis had to be limited to mid-cavity segments. Although normal segmental Ecc by echo predicted absence of scar in the corresponding segment by CMR, the correlation between VVI and CMR assessments of Ecc in mid-cavity segments was limited. Based on these results, although VVI echocardiographic assessment of regional Ecc in DMD was feasible, it does not appear to be an adequate surrogate in DMD for CMR with MTT, which is presently regarded as the gold standard for myocardial Ecc [13,35].

With respect to electrocardiography, although there may be clues in the 12-lead electrocardiogram suggestive of more advanced cardiac disease and scar, we were unable to identify a specific predictor relative to CMR findings, highlighting the importance of accurate and feasible cardiac imaging for this purpose.

## Conclusions

The relationship between scar and Ecc in DMD is complex. Among myocardial segments with depressed Ecc, scar prevalence was much higher in inferior, inferolateral, and anterolateral segments, indicating a regionally dependent association between abnormal Ecc and scar. As a result, LV septal segments are more likely to develop abnormal Ecc without scar in DMD, whereas abnormal Ecc is commonly associated with scar in LV free wall segments. With respect to echocardiography, although normal segmental Ecc by VVI is associated with absence of scar, VVI assessment of abnormal regional echocardiographic Ecc was only weakly associated with CMR Ecc in DMD.

## Abbreviations

CMR:  cardiovascular magnetic resonance; DMD:  Duchenne muscular dystrophy; Ecc:  circumferential strain; ECG:  electrocardiogram; HARP:  harmonic phase; IQR:  interquartile range; LGE:  late gadolinium enhancement; LV:  left ventricle; LVEDV:  left ventricular end-diastolic volume; LVESV:  left ventricular end-systolic volume; MTT:  myocardial tissue tagging.

## Competing interests

The authors declare that they have no competing interests.

## Authors' contributions

KCB played an important role in the conception/design of the study, acquired the CMR data, performed the primary analysis of CMR strain and scar data, and drafted the manuscript. DP and MS also performed analysis of CMR data and revised the manuscript critically for important intellectual content. YD performed analysis of the echocardiographic data and provided a critical review of the manuscript. TC and DD were involved in the conception and design of the study, performed neurologic evaluations of the study participants, and provided a critical review of the manuscript for important intellectual content. WRT was involved in the conception and design of the study, both acquired and analyzed the echocardiographic data, and provided a critical review of the manuscript. All authors have reviewed and approved the final manuscript.
